# Crystal Structure of the ORP8 Lipid Transport ORD Domain: Model of Lipid Transport

**DOI:** 10.3390/cells12151974

**Published:** 2023-07-31

**Authors:** Andrea Eisenreichova, Martin Klima, Midhun Mohan Anila, Alena Koukalova, Jana Humpolickova, Bartosz Różycki, Evzen Boura

**Affiliations:** 1 Institute of Organic Chemistry and Biochemistry AS CR, v.v.i., Flemingovo nam. 2., 166 10 Prague, Czech Republic; andrea.eisenreichova@uochb.cas.cz (A.E.); martin.klima@uochb.cas.cz (M.K.); alena.koukalova@uochb.cas.cz (A.K.); jana.humpolickova@uochb.cas.cz (J.H.); 2 Institute of Physics, Polish Academy of Sciences, Al. Lotników 32/46, 02-668 Warsaw, Poland; midhun@ifpan.edu.pl (M.M.A.); rozycki@ifpan.edu.pl (B.R.)

**Keywords:** lipid transport, ORD, ORP8, PS, PI4P, plasma membrane, ER

## Abstract

ORPs are lipid-transport proteins belonging to the oxysterol-binding protein family. They facilitate the transfer of lipids between different intracellular membranes, such as the ER and plasma membrane. We have solved the crystal structure of the ORP8 lipid transport domain (ORD8). The ORD8 exhibited a β-barrel fold composed of anti-parallel β-strands, with three α-helices replacing β-strands on one side. This mixed alpha–beta structure was consistent with previously solved structures of ORP2 and ORP3. A large cavity (≈1860 Å^3^) within the barrel was identified as the lipid-binding site. Although we were not able to obtain a lipid-bound structure, we used computer simulations based on our crystal structure to dock PS and PI4P molecules into the putative lipid-binding site of the ORD8. Comparative experiments between the short ORD8^ΔLid^ (used for crystallography) and the full-length ORD8 (lid containing) revealed the lid’s importance for stable lipid binding. Fluorescence assays revealed different transport efficiencies for PS and PI4P, with the lid slowing down transport and stabilizing cargo. Coarse-grained simulations highlighted surface-exposed regions and hydrophobic interactions facilitating lipid bilayer insertion. These findings enhance our comprehension of ORD8, its structure, and lipid transport mechanisms, as well as provide a structural basis for the design of potential inhibitors.

## 1. Introduction

Lipid transport between various membranous intracellular organelles occurs by either vesicular transport or the action of a conserved protein family specialized in lipid transportation [1]. Lipid transfer at membrane contact sites, such as the ER–plasma membrane contact site, is facilitated by oxysterol-binding protein (OSBP)-related proteins (ORPs). This mechanism ensures efficient lipid transport from the site of lipid synthesis (ER) to target membranes (plasma membrane, Golgi, mitochondria), while also maintaining the appropriate lipid composition of intracellular membranes [2,3]. Consequently, tight control of this process is crucial.

There are several human ORP proteins that are composed of multiple domains, with the majority containing a lipid-binding domain and a lipid transport domain connected by a flexible linker. Most human ORP proteins contain an N-terminal pleckstrin homology (PH) domain and a C-terminal lipid transport domain. The ORP8 protein has an additional C-terminal transmembrane helix [4]. The PH domain recognizes and binds the target membrane, while the C-terminal transmembrane helix anchors ORP8 to the donor (ER) membrane. This configuration represents the active state of the protein, facilitating lipid cargo transport by the ORD8 domain [5]. The directionality of this process is determined by the lipid composition of the membrane, whereby only target membranes with a particular composition are recognized by the PH domain [5,6,7], as well as by the concentration of the lipid cargo itself. However, certain ORPs posses the ability to transport lipids against their concentration gradient. In the case of ORP8, the cargo lipid is phosphatidylserine (PS), which is synthesized in the ER but needs to be transported to the plasma membrane. Such an uphill process requires energy. In this scenario, the “fuel” is not direct ATP hydrolysis but rather the concentration gradient of the lipid phosphatidylinositol 4-phosphate (PI4P), generated at the plasma membrane [8,9,10]. PI4P is transported from the plasma membrane and is exchanged for PS at the ER, followed by hydrolysis by the Sac1 phosphatase to ensure the non-reversibility of this transport [5,11,12]. Importantly, this process is conserved from yeast to humans [13,14].

Although the functional role of ORP8 is understood, its structural mechanism remains incompletely characterized. However, the lipid transport mechanism of the closely related protein ORP2, which transports both cholesterol and PI(4,5)P_2_, has recently been elucidated [15]. Cholesterol-loaded ORP2 binds to the plasma membrane, where cholesterol is exchanged for PI(4,5)P_2_. This process is coupled with the tetramerization of ORP2 [15]. Subsequently, PI(4,5)P_2_ is transported to the endosomal compartment, where it is likely hydrolyzed by a 5-phosphatase [16]. Importantly, cholesterol and PI(4,5)P_2_ occupy the same lipid-binding pocket, meaning that PI(4,5)P_2_ and cholesterol cannot simultaneously bind to the ORP2 protein. Additionally, one region, located above the lipid-binding pocket, known as the “lid”, must undergo movement during cargo lipid loading and unloading. It is highly likely that these two features are conserved in the case of ORP8.

Apart from the metazoan ORP8 (and others also including ORP5/10), yeast proteins Osh6/7 also have similar structural features and it is supposed that they share the mechanism of the PS/PI4P exchange [13]. Both Osh6/7 consist merely from the lipid transfer domain [13,17]. However, the cellular localization of Osh6 is determined by the adaptor protein Ist2 [18,19], a membrane tether that is anchored in the ER and recognizes PI(4,5)P_2_ in the PM [20]. Thus, this protein complex functionally resembles the multidomain architecture of ORP8.

At the molecular level, it is again the N-terminal lid that contributes to the regulation of the Osh6 transport function. The lid has been shown to shield the basic surface of the protein that enables the recognition of the acidic membrane surface [21]. In our recent work, we show that it is the cargo that imposes changes in the dynamics of the lid and thus contributes to the recognition of the cargo-specific target membrane [22].

To gain a more detailed structural understanding of the mechanism behind ORP8 lipid transport, we focused on determining the crystal structure of its ORD domain (ORD8) while bound to a cargo lipid. However, despite years of efforts, we have only been successful in elucidating the structure of unliganded ORD8. Nevertheless, we utilized a combination of our crystal structure data and computer simulations to gain insights into the binding of phosphatidylserine (PS) and phosphatidylinositol 4-phosphate (PI4P) to the ORP8 protein. This integrated approach also enabled us to characterize the binding mode of ORP8 to the membrane. Additionally, fluorescence-based lipid-transport experiments highlighted the importance of the lid.

## 2. Materials and Methods

### 2.1. Protein Expression and Purification

The genes encoding ORD8 (residues 376–791) and ORD8^ΔLid^ (residues 406–791) were cloned into a modified pHIS2 vector containing N-terminal His6x-tag followed by SUMO tag and expressed using our usual protocols [23,24]. Briefly, *E. coli* BL21 Star cells were transformed by the expression plasmids and the cells were grown in Luria–Bertani medium at 37 °C until OD_600_ reached 0.6–0.8. Expression was induced with IPTG at a 0.5 mM final concentration, and the cells were cultivated overnight at 18 °C. The next day, they were harvested by centrifugation, resuspended in lysis buffer (50 mM Tris (pH 8), 300 mM NaCl, 20 mM imidazol, and 3 mM β-mercaptoethanol) and lysed by sonication. Upon clearing the lysate by centrifugation, the supernatant containing His-SUMO-ORD8 was incubated with Ni-NTA beads for 60 min. The beads were washed with lysis buffer, and the fusion protein was eluted with lysis buffer supplemented with 300mM imidazol. The His-SUMO tag was cleaved off by the Ulp1 protease. The proteins were further purified by ion-exchange chromatography on a HiTrap SPHP column (Cytiva) in 20 mM HEPES (pH 7.0), 3 mM β-mercaptoethanol with 50mM-1M NaCl gradient, and by size exclusion chromatography (SEC) on a HiLoad 16/600 Superdex75 pg column (Cytiva) in SEC buffer (20mM Tris (pH 7.4), 300 mM NaCl 3 mM β-mercaptoethanol, 10% glycerol). The proteins were concentrated to 5 mg/mL and stored in −80 °C until needed.

### 2.2. Crystallization and Data Collection

For crystallization experiments, ORD^ΔLid^ was transferred to buffer composed of 20mM HEPES (pH 7.0), 100 mM NaCl, and 3mM dithiothreitol and concentrated to 3 mg/mL. Screening experiments were performed using the sitting drop vapor diffusion technique in a 96-well plate. Drops were created using the Mosquito crystallization robot (SPT Labtech, Melbourn, UK) by mixing 150 nL of protein solution with 150 nL well solution, giving rise to the initial microcrystals in one day. The crystallization conditions were further optimized, and thin, plate-like crystals were obtained in two days from drops created by mixing 75 nL of protein solution with 225 nL well solution (0.1M HEPES (pH 7.0) and 15% PEG 20000) and equilibrated against 70 μL of well solution. The crystals were cryoprotected in mother-liquor supplemented with 20% glycerol and flash-frozen in liquid nitrogen.

The crystallographic dataset was collected from a single crystal on the BL14.2 beamline at the BESSY II electron storage ring operated by the Helmholtz-Zentrum Berlin [25]. The dataset was collected at the temperature of 100 K using the wavelength of 0.9184 Å. The crystals diffracted to 2.5 Å resolution and belonged to the P2_1_2_1_2 space group. The data were integrated and scaled using XDS [26]. The merged data were corrected for diffraction anisotropy by ellipsoidal truncation and anisotropic scaling using the Diffraction Anisotropy Server (https://srv.mbi.ucla.edu/Anisoscale/ (accessed on 8 March 2023)). The crystal structure was solved by molecular replacement using an in silico model generated with AlphaFold v2.0 [27] as a search model. The initial model was obtained with Phaser v2.8.3 [28]. The model was further improved using automatic model refinement with the phenix.refine tool [29] from the Phenix package v1.20.1-4487 [30] and manual model building with Coot v0.9.8.7 [31]. Statistics for data collection and processing, structure solution, and refinement were calculated with the phenix.table_one tool and are summarized in Table 1. Structural figures were generated with the PyMOL Molecular Graphics System v2.5.4 (Schrödinger, LLC, New York, NY, USA). The atomic coordinates and structural factors were deposited in the Protein Data Bank (https://www.rcsb.org (accessed on 8 March 2023)) under the PDB accession code 8P7A.

### 2.3. Lipids and Other Chemicals

All lipids were purchased from Avanti Polar Lipids (Alabaster, AL, USA) and were used without further purification. Atto488-labeled DOPE was obtained from ATTO-TEC (Siegen, Germany), and the lipid tracer DiD and other basic chemicals were purchased from Sigma-Aldrich (St. Louis, MO, USA).

### 2.4. LUV Formation

LUVs were prepared by extrusion as described elsewhere [32]. Briefly, lipids in organic solvents were mixed in the desired ratio so that the final lipid concentration in the LUVs was 1 mM. The organic solvents were evaporated in a stream of nitrogen and kept in a vacuum chamber for at least one hour. Subsequently, the lipid films were resuspended in LUV buffer (40 mM imidazole (pH 7.4), 150 mM NaCl, 3 mM beta-mercaptoethanol, 1 mM EDTA), and 50 nm diameter LUVs were prepared using an extruder with a membrane of appropriate pore size.

### 2.5. Kinetics Assays

The kinetics data were acquired in a short, 200 s measurement before adding the transporter, followed by a longer measurement (20 min) after its addition. The concentration of ORD8 constructs was 250 nM, whereas the biosensors C2_Lact_-CFP and SidC-Atto488 were present in the total volume of 200 μL at concentrations of 50 nM and 100 nM, respectively. For the PS transport assays, 10 µL of donor LUVs, composed of 91 mol % POPC, 5 mol % diphytanoyl-PG, and 4 mol % POPS, labeled with DiD, were mixed with C2_Lact_-CFP, the LUV buffer, and either 0 µL or 40 µL of unlabelled LUVs with different lipid compositions (POPC or POPC/PI4P (5 mol %)). For the PI4P transport assays, 10 µL of donor LUVs comprising 97 mol % POPC and 3 mol % PI4P labeled with DiD were combined with SidC-Atto488, the LUV buffer, and either 0 µL or 40 µL of unlabelled LUVs composed of various lipid mixtures (DOPC or DOPC/POPS (20 mol %)). The labeling of LUVs with DiD was done at a DiD/lipid ratio of 1/10,000. All the FCCS experiments were carried out in at least three independent replicates to ensure reproducibility.

### 2.6. Microscopy

The FCCS experiments were conducted under a Leica SP8 confocal microscope (Leica, Mannheim, Germany) equipped with a high numerical aperture water objective (63×, N.A. = 1.2), a set of synchronizable pulsed lasers, and sensitive hybrid HyD detectors. In our experiments, we utilized the 640 nm line of the white light laser (Coherent, Inc., Santa Clara, CA, USA) to excite DiD, and the 440 nm and 470 nm diode laser heads (Picoquant, Berlin, Germany) to excite CFP and Atto488, respectively. The pair of lasers (440/640 and 470/640) alternated in the pulsed interleaved excitation (PIE) mode with overall repetition frequencies of 40 MHz and 20 MHz, respectively. The PIE mode was employed to apply temporal filtering of photon arrival times and spectral information to eliminate bleed-through. The acquired data were correlated and analyzed using custom scripts in Matlab (Mathworks, Natick, MA, USA).

### 2.7. All-Atom MD Simulations

Using VMD [33], we superimposed the ORP8 ORD structure with structures of POPS- and PI4P-loaded Osh6 [13,17] (PDB entry codes 4B2Z and 4PH7, respectively), which provided us with preliminary structural models of POPS- and PI4P-loaded ORP8 ORD. Using the input generator on the CHARMM-GUI website [34], each of the two structural models was solvated in a cubic box with the side length of 9.3 nm, and then sodium and chloride ions were added to neutralize the systems and to reach a physiological ion concentration of 150 mM. The energy of the solvated systems was minimized in 10,000 conjugate-gradient steps. Then, the systems were equilibrated in two subsequent steps: (i) 0.5 ns MD simulations at constant volume and temperature T = 303 K and with harmonic restraints on the coordinates of heavy atoms of the protein and lipid and (ii) 10 ns unrestrained MD simulations at a pressure p = 1 atm and a temperature of T = 303 K.

The MD simulations were performed using NAMD 2.14 with a CHARMM36 force field and the TIP3P model for water molecules [35,36,37]. The temperature was kept at T = 303 K through the Langevin thermostat with a damping coefficient of 1/ps. Pressure was maintained at p = 1 atm using the Langevin piston Nose-Hoover method with a damping timescale of 25 fs and an oscillation period of 50 fs. Short-range non-bonded interactions were cutoff smoothly between 1 and 1.2 nm. Long-range electrostatic interactions were computed using the particle mesh Ewald method with a grid spacing of 0.1 nm. Simulations were performed with an integration time step of 2 fs. For each of the two simulation systems (i.e., POPS- and PI4P-loaded ORP8 ORD models), we performed a production run of 100 ns. Frames were saved every 100 ps. The simulation trajectories were visualized and analyzed using VMD [33]. Contacts between the protein domain and the lipid were determined using a standard distance criterion: if the distance between any atom of a given amino acid residue and any atom of the lipid is smaller than 0.45 nm, then this amino acid residue is deemed to be in contact with the lipid.

### 2.8. Coarse-Grained MD Simulations

The system for coarse-grained MD simulations was set up in the following way using the Martini maker on the CHARMM-GUI input generator website [38]. A bilayer with lateral dimensions of 12 nm by 12 nm was formed of 352 POPC and 88 POPS lipids (i.e., with a 4:1 molar ratio). The ORP8 ORD structure was placed about 4 nm above the lipid bilayer. The system of lipids and protein was placed in a cuboid box and solvated. Sodium and chloride ions were added to neutralize the systems and to reach a physiological ion concentration of 150 mM. The simulation system was coarse grained within the framework of the Martini 3 model with an elastic network (ELNEDIN) applied to protein beads [39].

The initial systems for MD simulations were energy-minimized using a conjugate gradient method and then equilibrated in a standard procedure using input files generated by the Martini maker on the CHARMM-GUI input generator website. The coarse-grained MD simulations were performed using Gromacs 2020.2 and the Martini 3 force field [39,40]. Periodic boundary conditions were applied. Temperature and pressure were kept constant at T = 303 K and p = 1 bar, respectively, using the velocity-rescaling thermostat and the Parrinello–Rahman barostat [41,42]. Non-bonded interactions were treated with the Verlet cutoff scheme. The cutoff for Van der Waals interactions was set to 1.1 nm. Coulomb interactions were treated using the reaction-field method with a cutoff of 1.1 nm and dielectric constant of 15. The integration time step was set to 20 fs. Two independent production runs of 100 µs were performed. Frames were saved every 1 ns. The simulation trajectories were post-processed using MDVWhole to treat the periodic boundary conditions and visualized using VMD [33].

## 3. Results

### 3.1. Overall Structure and Functional Characterization of the ORP8 Lipid Transport Domain

**Figure 1:**
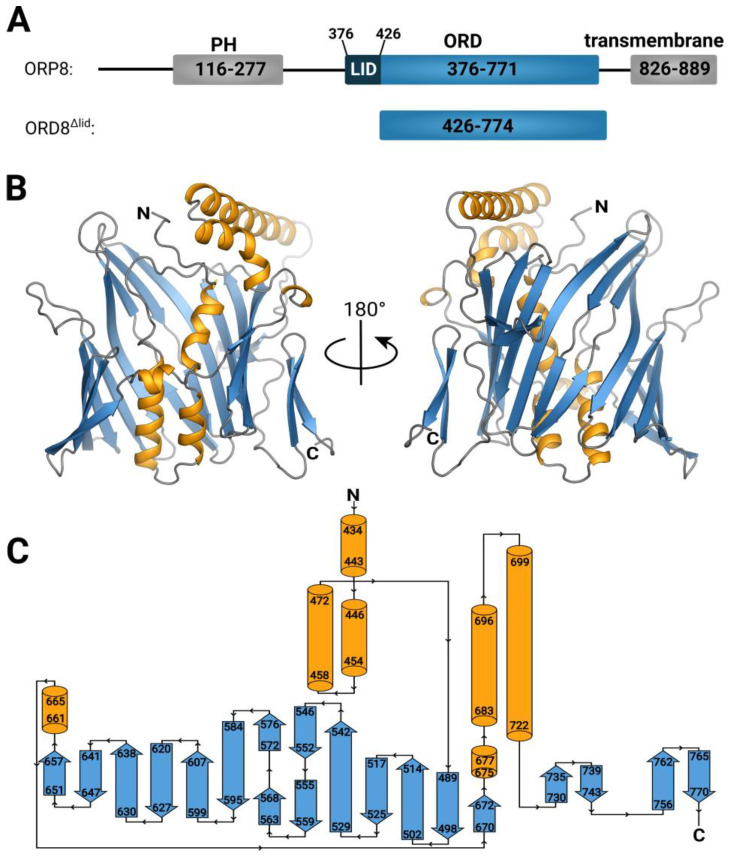
Crystal structure of the ORD8 domain. (**A**) Domain organization of the ORP8 protein. (**B**) Overall crystal structure of the ORD8 domain. (**C**) Topology plot of the ORD8 domain.

The structure was solved by molecular replacement using an AlphaFold model. The RMSD between the model and the refined crystal structure was 1.44 Å, and the structure fitted the 2mFo-DFc composite omit map well (Figure S1). The overall fold is reminiscent of a β-barrel composed of anti-parallel β-strands. Compared to the well-studied β-barrel structure, the GFP [43], the ORD8 domain is composed of more β-strands (19 in total), whereas GFP is composed of 11 β-strands. However, in the case of ORD8, in one side of this β-barrel, the β-strands are replaced by three α-helices (Figure 1). The fold is therefore actually a mixture of alpha and beta. This observation is consistent with the previously reported structures of ORP2 and ORP3 [15,44,45] that exhibit similar fold with RMSDs at 5.27 Å and 5.32 Å, respectively (Figure S2).

**Figure 2:**
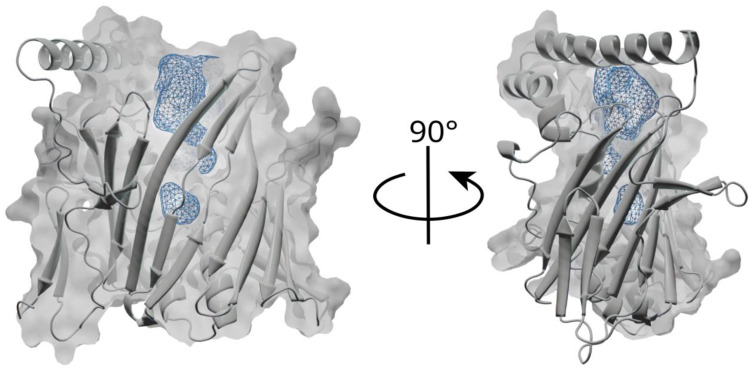
Lipid-binding cavity of the ORD8 domain. Sliced surface of the ORD8 domain shown in the surface and cartoon representation (grey) with the highlighted lipid-binding cavity shown in blue mesh. The cavity was calculated using the CavitOmiX (v. 1.0, 2022, Innophore GmbH, Graz, Austria) Pymol plugin.

**Figure 3:**
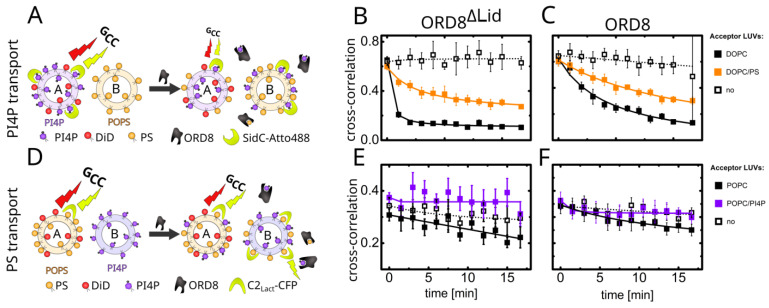
PS and PI4P transport assays by ORP8 ORD constructs. (**A**) Scheme of the PI4P transport assay. LUVs A contain DiD and PI4P, made visible by its biosensor SidC-Atto488. LUVs B are unlabeled. The cross-correlation (here plotted as *G*_cc_(0)/G_R_(0)) arose from the mutual motion of DiD and SidC-Atto488. Upon the addition of the transporter, PI4P moved to LUVs B and the mutual motion of the two fluorophores, and consequently *G*_cc_ were lowered. If LUVs B contained PS, the two cargo lipids competed for the binding site of the protein. (**B**,**C**) Kinetics of the PI4P transport accomplished by ORD8^ΔLid^ and ORD8, respectively. The LUVs B were composed of DOPC (black line) or DOPC/POPS (20 mol %) (orange line), or were missing (dotted line). (**D**) Scheme of the PS transport assay. LUVs A contained DiD and PS visualized by its biosensor C2_Lact_-CFP. LUVs B were unlabelled. The cross-correlation *G*_cc_ arose from the mutual motion of DiD and C2_Lact_-CFP. Upon the addition of the transporter, PS moved to LUVs B and the mutual motion of the two fluorophores, and consequently *G*_cc_ were lowered. If LUVs B contained PI4P, the two cargo lipids competed for the binding site of the protein. (**E**,**F**) Kinetics of the PS transport accomplished by ORD8^ΔLid^ and ORD8, respectively. The LUVs B were composed of POPC (black line) or POPC/PI4P (5 mol %, violet line), or were missing (dotted line). The data were fitted by a hyperbola, and the error bars represent standard error. The experiments were conducted independently at least twice to ensure the reproducibility of the observed trends.

The structure also revealed a rather large cavity (its volume was estimated using spaceball [46] to be approximately 1860 Å^3^) in the middle of the barrel (Figure 2). This cavity, consistently with the previously reported structure of Osh6 [13,17], represents the lipid-binding site. Despite all our efforts, we were not able to obtain the structure with a lipid bound. This observation highlights the importance of the lid for the stable binding of lipids by the ORD domain. The full-length (including the lid) ORD domain never produced any crystals. This could be attributed to the lid’s flexibility, which probably impeded crystallization. However, we were able to compare the lipid transport properties of the crystallized domain ORD8^ΔLid^ (shorter construct without a lid) and the full-length transport domain ORD8 (longer, lid containing, construct). In our experiments, based on fluorescence cross-correlation spectroscopy (FCCS), the lipid-donating, fluorescently labeled large unilamellar vesicles (LUVs) and the non-labeled acceptor vesicles were mixed. The addition of a fluorescently labeled biosensor for the detection of a cargo lipid (either SidC for PI4P or the C2 domain of lactadherin for PS) resulted in a double labeling of the donor LUVs. This, in turn, led to a high cross-correlation between the two fluorescence signals, specifically in the FCCS read-out parameter we monitored during the transport, marked as G_cc_(0)/G_R_(0) or Gcc for simplicity. Upon the addition of the transporter, the cargo, followed by its biosensor, was driven to the acceptor LUVs, which was accompanied by a drop in G_cc_ (Figure 3A,D).

The transport of PS and PI4P from donor LUVs to acceptor LUVs of various kinds is summarized in Figure 3. If no acceptor LUVs were present in the systems, no transport was observed in any instances, as the extracted cargo from the donor membrane was not within the dynamic range of the biosensors’ response (Figure 3B,C,E,F, dotted curves). However, when competing cargo non-containing LUVs were added, the extracted ligand was able to be deposited onto the target membrane, resulting in the transport of both PS and PI4P (Figure 3B,C,E,F, black curves). In the case of PI4P transport (Figure 3B,C, black curves), PI4P was transported to a large extent. The observed transport was faster when facilitated by the ORD8^ΔLid^ domain, as the more open form of the protein provided less stabilization for the cargo inside its cavity. In contrast to PI4P, PS transport (Figure 3E,F, black curves) occurred to a smaller extent and was also slightly more pronounced for the ORD8^ΔLid^ protein. These results imply that the lid slows down the transport of both cargoes, as it is likely involved in the stabilization of the ligand.

If the acceptor LUVs contain a competing cargo, the transport of PS is completely inhibited by PI4P (Figure 3E,F, violet curves), and the transport of PI4P is slowed down (Figure 3B,C, orange curves). However, in the case of Osh6 [12], facilitation of PI4P transport was observed when the acceptor LUVs contained an excess of PS. This was not the case for either of the ORD8 constructs tested.

### 3.2. Lipid Binding Mode of the ORP8 ORD Domain

**Figure 4:**
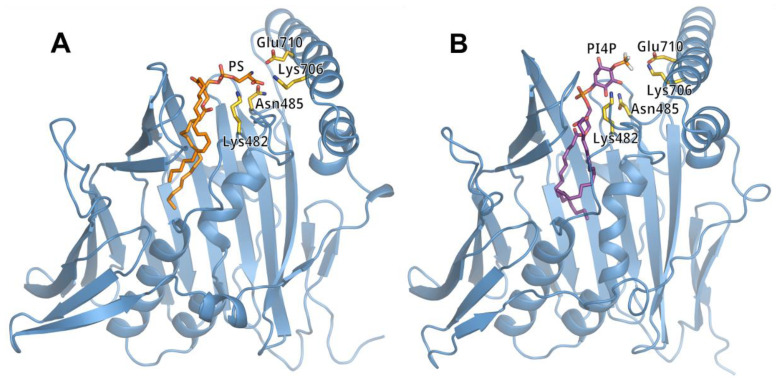
Lipid-binding mode predicted in MD simulations. Simulation snapshots of ORD8 domain loaded (**A**) by POPS and (**B**) by PI4P. The ORD domain is shown in blue in the cartoon representation. The lipids are shown in the stick representation. The key amino acid residues identified in the MD simulations to interact with the lipid headgroup (Lys482, Asn485, Lys706, and Glu710) are also shown in the stick representation. The snapshots were taken at the last nanosecond of the MD trajectories.

We performed molecular dynamics (MD) simulations to dock POPS and PI4P molecules into the putative lipid-binding site of the ORD8 domain (Figure 4, Figures S3 and S4). The structural models of POPS-loaded and PI4P-loaded ORD8, as obtained from the MD simulations, resembled the corresponding structures of Osh6 [13,17]. However, in contrast to the structures of POPS- and PI4P-loaded Osh6, which contain the N-terminal lid, in the ORD8 simulation structures, we found both hydrocarbon chains of the lipid inserted deeper into the hydrophobic core of the protein. Analysis of the MD trajectories showed that the secondary structure elements that contacted the lipid hydrocarbon chains were α helices 434–443 and 458–472; loop 472–489; and β strands 529–542, 546–552, 572–576, and 584–595 (Figure 1C, Figure 4 and Figure S3A,C). Our MD trajectory analysis also revealed that the amino acid residues that most frequently make contacts with the lipid headgroups were Lys482, Asn485, Lys706, and Glu710 (Figure 4 and Figure S3B,D). Interestingly, these residues were conserved in Osh6. Specifically, Lys482 and Asn485 in ORP8 corresponded to Lys126 and Asn129 in Osh6. And Lys706 and Glu710 in ORP8 corresponded, respectively, to Lys351 and Glu355 in Osh6. Moreover, Lys482 and Lys706 often formed hydrogen bonds with the lipid headgroups in the cases of both POPS and PI4P.

**Figure 5:**
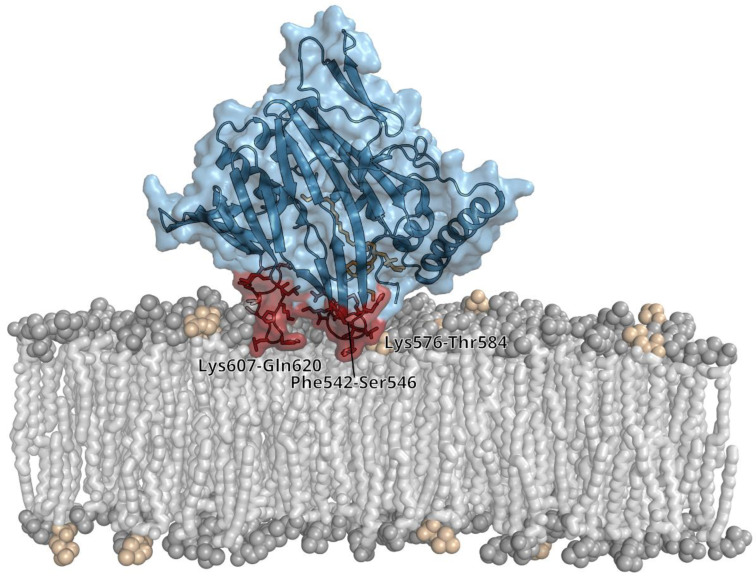
Membrane-binding mode of ORP8 ORD obtained in coarse-grained MD simulations.

We also performed coarse-grained MD simulations of ORD8 interacting with a bilayer of POPC and POPS lipids (Figure 5). The molar ratio of POPC and POPS was 4:1. At the time scale of 100 µs, we were able to observe multiple events of binding and unbinding of ORD8 to/from the lipid bilayer in the MD trajectories (Figures S5 and S6). Importantly, analysis of the MD trajectories revealed that the ORD8 binding was mediated by a surface-exposed site that was inserted into the lipid bilayer. The predicted site of membrane insertion involved loops 542–546, 576–584, and 607–620 (Figure 5, Figures S5 and S6). In particular, the amino acid residues that submerged into the hydrophobic core of the lipid bilayer were Tyr543, Leu579, Tyr580, Phe611, and Leu612. At the same time, many adjacent amino acid residues (including Lys576 and Lys609) made contact with lipid headgroups. Interestingly, in this binding pose, ORD8 was oriented with its putative lipid-binding site towards the lipid bilayer surface (Figure 5).

## 4. Discussion

The delicate homeostatic mechanism of lipid transport by ORPs is well described, yet how it is achieved is not understood at the molecular level. We hypothesize that the PH domain acts as an auto-inhibitory mechanism that blocks the function of the ORD domain, and conversely, this interaction impedes the interaction of the PH domain with the plasma membrane. We speculate that PS binding alters the conformation of the ORD domain in such a way that the PH domain can establish contact with the plasma membrane. Conversely, PI4P/PI(4,5)P_2_ binding to the PH domain decreases the PS affinity of the ORD, favoring cargo release at the plasma membrane. At the ER, the PI4P lipid is released from the ORD and becomes immediately hydrolyzed by Sac1 phosphatase, and a new cargo, PS, is loaded. PS is then transported against its gradient towards the plasma membrane. The PS induced conformational change could initiate the detachment of the ORD domain from the ER, and the interaction with the PH domain could be the driving force behind the attraction of the ORD domain to the target membrane. These theories should be tested in order to have a deeper structural understanding of lipid transport processes in eukaryotic cells. Recently, several inhibitors of lipid transport domains were reported [47]. Structure-based inhibitor design is being increasingly utilized by us and others [48,49,50,51,52] in the development of potent inhibitors. The crystal structure of ORD8 will provide the necessary structural foundation.

## Figures and Tables

**Table 1:** Data collection and refinement statistics. Statistics for data collection and processing, structure solution, and refinement of the crystal structure of the ORD domain of human ORP8. The merged data were corrected for diffraction anisotropy by ellipsoidal truncation and anisotropic scaling, causing a drop in data completeness and in the number of reflections used for refinement. Resolution limits of the data along the reciprocal axes were 2.56, 3.26, and 4.06 Å. Numbers in parentheses refer to the highest resolution shell. R.m.s., root-mean-square.

Crystal		hORP8/ORD
PDB accession code		8P7A
**Data collection and processing**		
Diffraction source		BESSY 14.2
Detector		Dectris Pilatus 2M
Wavelength (Å)		0.9184
Space group		P 2_1_ 2_1_ 2
Cell dimensions	a, b, c (Å)	105.6 190.2 58.7
	α, β, γ (°)	90.0 90.0 90.0
Resolution range (Å)		46.17–2.56 (2.65–2.56)
No. of total reflections		504,392 (47,110)
Multiplicity		12.9 (12.3)
No. of unique reflections	uncorrected	38,978 (3814)
	anisotropy-corrected	21,645 (133)
Completeness (%)	uncorrected	99.17 (98.90)
	anisotropy-corrected	52.87 (3.39)
Mean I/σ(I)		9.52 (0.51)
Wilson B factor (Å^2^)		34.28
R-merge		0.2847 (4.747)
R-meas		0.2964 (4.952)
CC1/2 (%)		99.8 (49.5)
CC* (%)		100.0 (81.4)
**Structure solution and refinement**		
R-work (%)		21.90 (42.87)
R-free (%)		25.08 (42.08)
CC-work (%)		73.4 (67.1)
CC-free (%)		80.3 (88.7)
R.m.s. deviations	bonds (Å)	0.003
	angles (°)	0.59
Average B factor (Å^2^)		35.30
Rotamer outliers (%)		0.00
Clashscore		1.00
Ramachandran (%)	favoured	99.13
	allowed	0.87
	outliers	0.00

## Data Availability

The data were deposited in the PDB database under PDB ID: 8P7A.

## Supplementary Materials

The following supporting information can be downloaded at https://www.mdpi.com/article/10.3390/cells12151974/s1. Figure S1: Data supporting Results Section 3.1. Figure S2: Data supporting Results Section 3.1. Figure S3: Data supporting Figure 4—MD simulation results of POPS-loaded and PI4P-loaded ORD8. Figure S4: Data supporting Figure 4—MD simulation results of POPS-loaded and PI4P-loaded ORD8. Figure S5: Data supporting Figure 5—results of coarse-grained MD simulations (trajectory 1). Figure S6: Data supporting Figure 5—results of coarse-grained MD simulations (trajectory 2).
