# Supplementary material for: Crystal Structure of the ORP8 Lipid Transport ORD Domain: Model of Lipid Transport
Source: Cells. 2023 Jul 31;12(15):1974. doi: 10.3390/cells12151974 (PMC10417380; doi:10.3390/cells12151974)
Supplement: Supplementary file 1 [file cells-12-01974-s001.zip › cells-2536767-supplementary.pdf]

cells

Crystal structure of the ORP8 lipid transport ORD domain: model of lipid transport

Andrea Eisenreichova [1], Martin Klima [1], Midhun Mohan Anila [2], Alena Koukalova [1], Jana Humpolickova [1], Bartosz Różycki [2] and Evzen Boura [1,*]

[1] Institute of Organic Chemistry and Biochemistry AS CR, v.v.i., Flemingovo nam. 2., 166 10 Prague 6, Czech Republic
[2] Institute of Physics, Polish Academy of Sciences, Al. Lotników 32/46, 02-668 Warsaw, Poland
* Correspondence: to boura@uochb.cas.cz

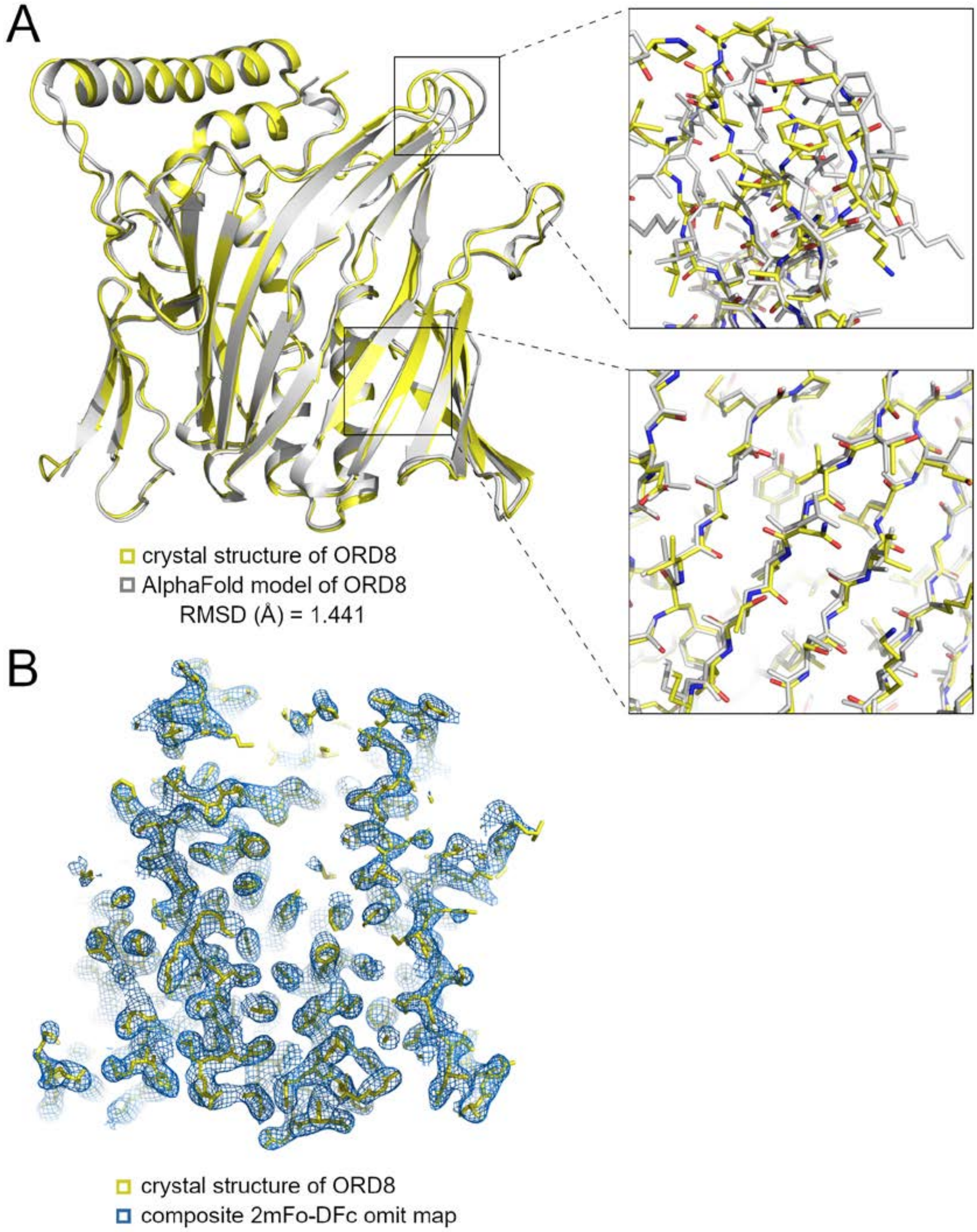

Figure S1. (**A**) Comparison of the crystal structure of the ORD8 domain (colored in yellow) with the AlphaFold model (colored in grey). (**B**) Representative electron density from a composite 2mFo-DFc omit map calculated using the phenix.composite_omit_map tool contoured at 1σ is shown in blue around the protein.

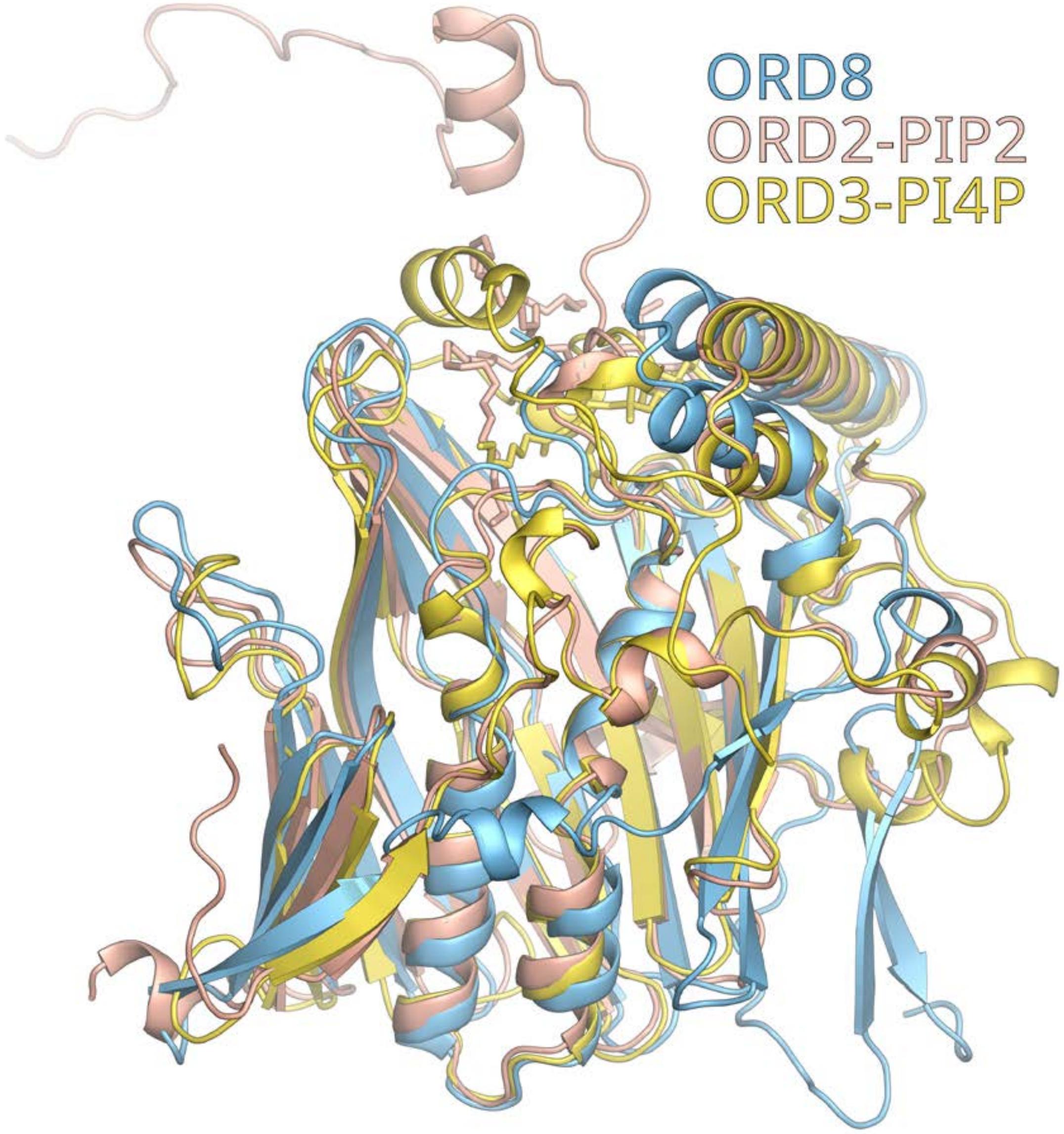

RMSD (Å)	ORD2	ORD3
ORD8	5.269	5.317

Figure S2. comparison of the ORD8 domain structure with structures of ORP2 and ORP3. ORD8 depicted in blue and PIP2-bound ORD2 domain in pink (PDB ID: 5ZM8) and PI4P-bound ORD3 in yellow (PDB ID:7DEI).

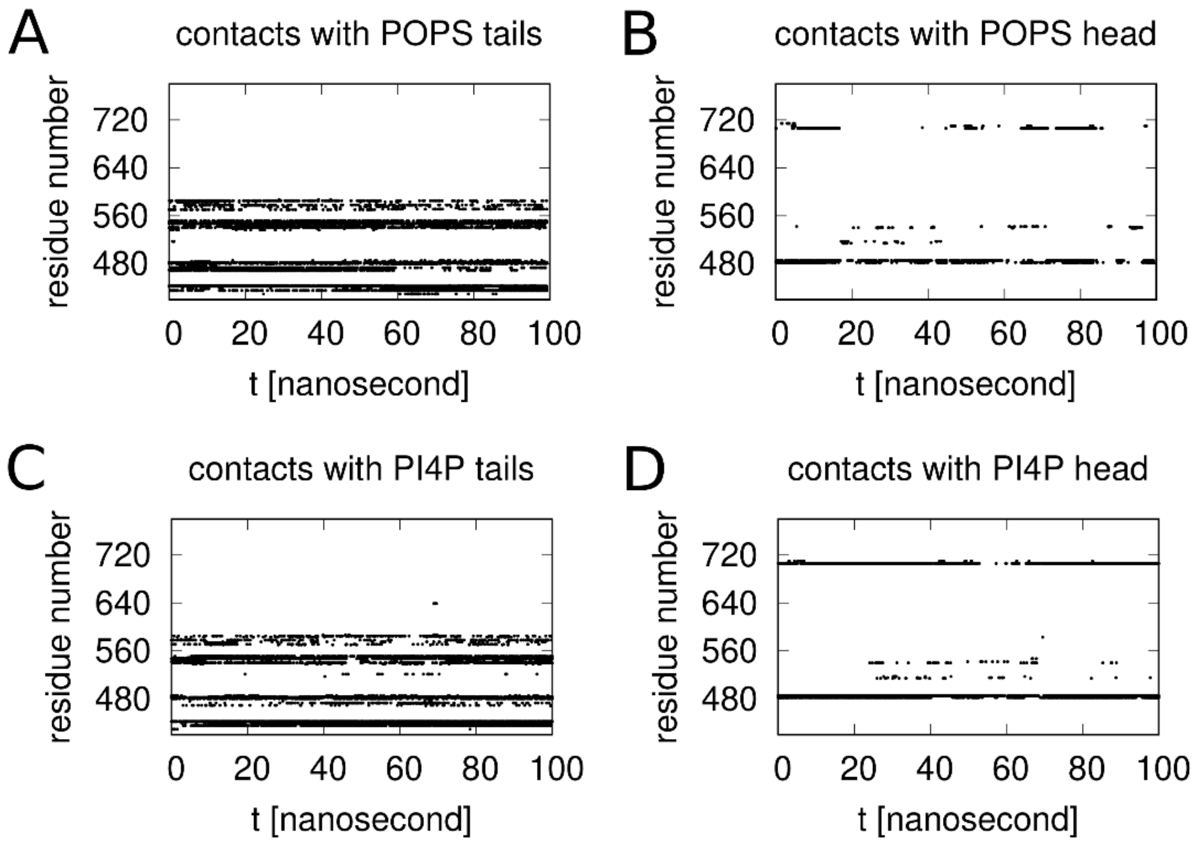

Figure S3. Data supporting Figure 4—MD simulation results of POPS-loaded (**A**,**B**) and PI4P-loaded (**C**,**D**) ORD8. (**A**,**B**) Contacts between ORP8 amino acid residues and POPS tails (**A**) and head (**B**) as a function of time. (**C**,**D**) Contacts between ORP8 amino acid residues and PI4P tails (**C**) and head (**D**) as a function of time.

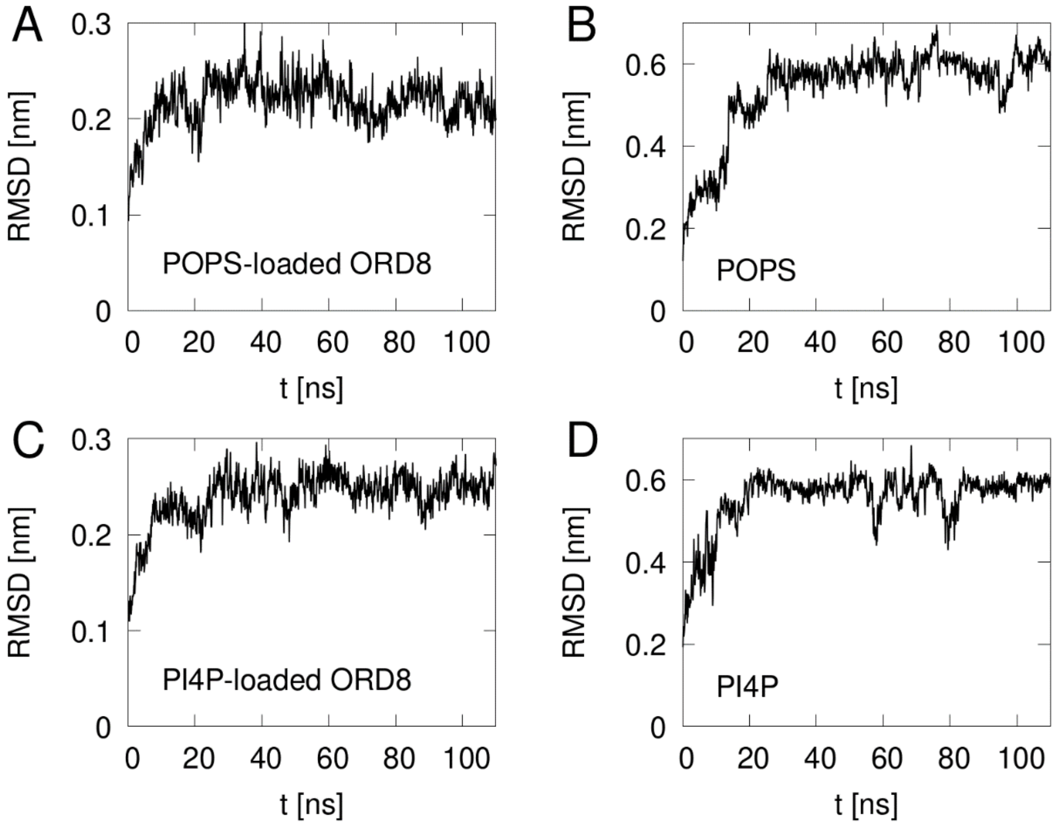

Figure S4. Data supporting Figure 4—MD simulation results of POPS-loaded (**A**,**B**) and PI4P-loaded (**C**,**D**) ORD8. (**A**,**B**) Root-mean-square deviation (RMSD) as a function of time for (**A**) the protein domain and (**B**) the POPS molecule. (**C**,**D**) RMSD as a function of time for (**C**) the protein domain and (**D**) the PI4P molecule.

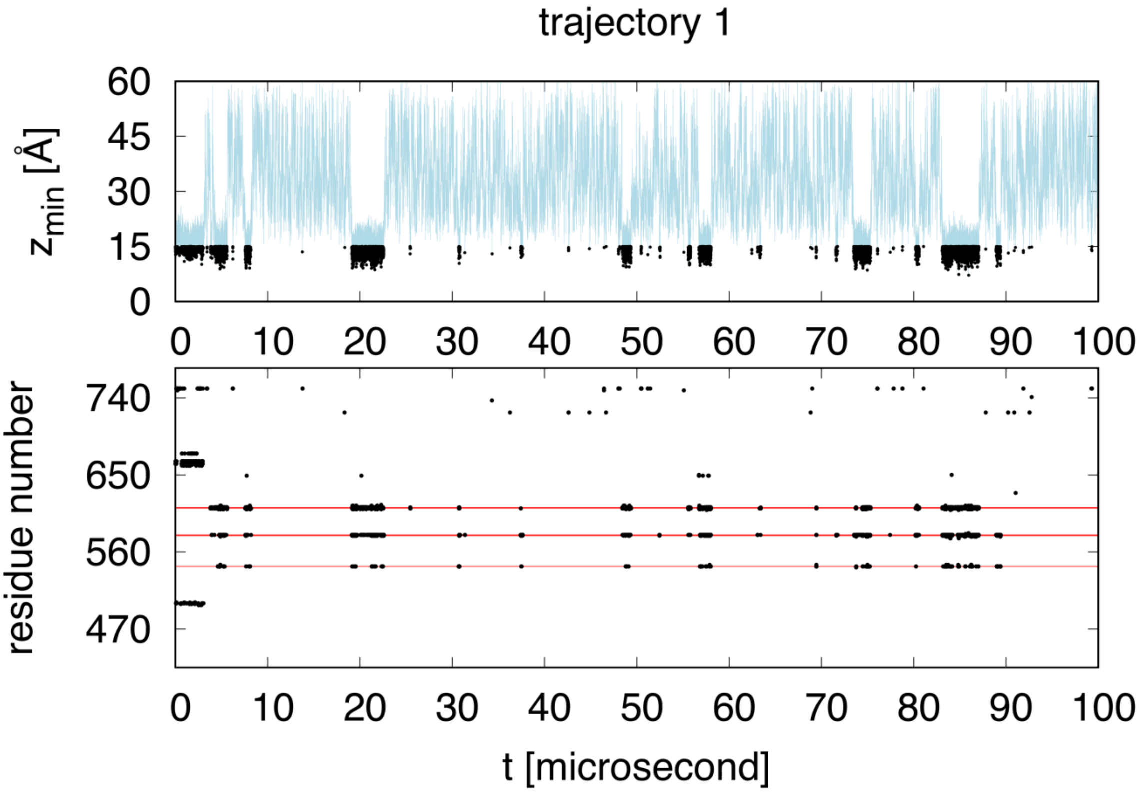

Figure S5. Data supporting Figure 5—Results of coarse-grained MD simulations (trajectory 1). The upper panel shows the minimal distance z_{min} between the lipid bilayer midplane and ORD8 amino acid residues as a function of time. The points in black indicate z_{min} < 1.5 nm where at least one amino acid residue of ORD8 is inserted into the hydrophobic core of the lipid bilayer. The lower panel shows which amino acid residues are inserted into the hydrophobic core of the lipid bilayer during the simulation run. The horizontal lines in red correspond to Tyr543, Leu579, Tyr580, Phe611 and Leu612.

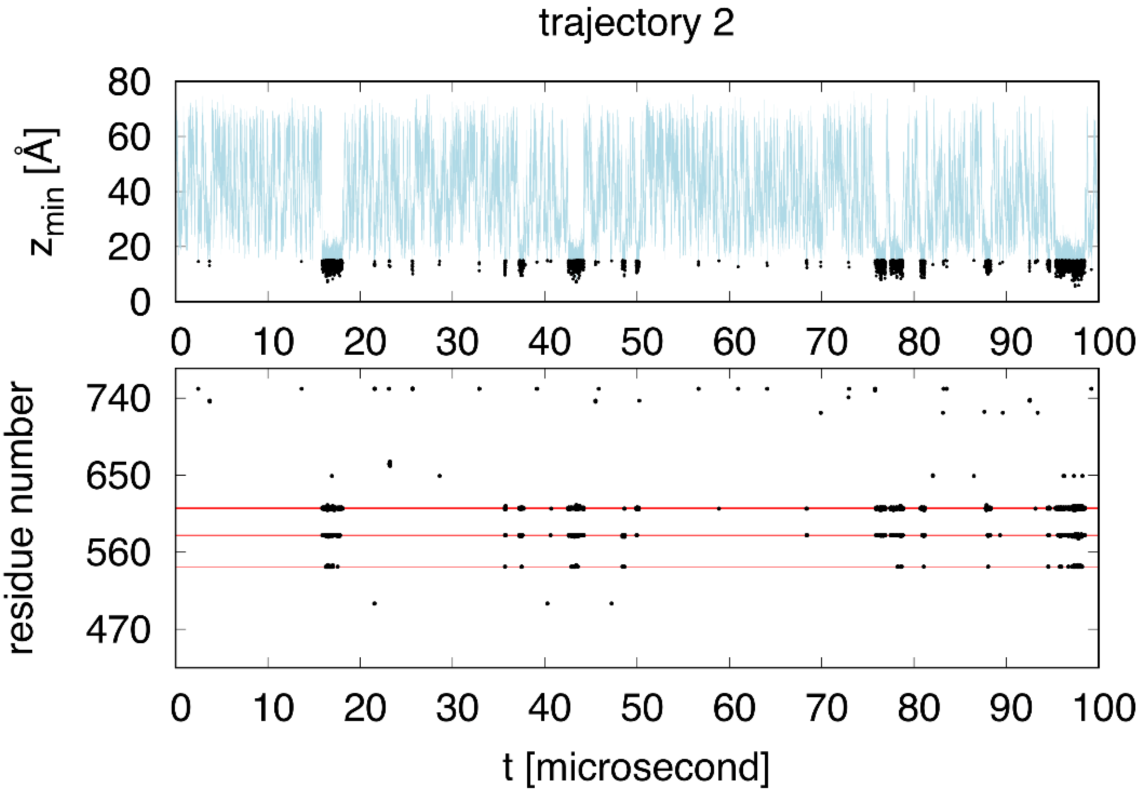

Figure S6. Data supporting Figure 5—Results of coarse-grained MD simulations (trajectory 2). Analogous to SI Figure 5 but obtained from an independent simulation run (trajectory 2).
